# Pullulanase with high temperature and low pH optima improved starch saccharification efficiency

**DOI:** 10.1038/s41598-022-26410-9

**Published:** 2022-12-19

**Authors:** Dandan Niu, Huihui Cong, Yanan Zhang, Nokuthula Peace Mchunu, Zheng-Xiang Wang

**Affiliations:** 1 Department of Biological Chemical Engineering, College of Chemical Engineering and Materials Science, Tianjin University of Science and Technology, Tianjin, 300457 China; 2 Agricultural Research Council, Biotechnology Platform, Private Bag X5, Onderstepoort, 0110 South Africa; 3 College of Biotechnology, Tianjin University of Science and Technology, Tianjin, 300457 China

**Keywords:** Biological techniques, Biotechnology, Microbiology

## Abstract

Pullulanase, a starch debranching enzyme, is required for the preparation of high glucose/maltose syrup from starch. In order to expand its narrow reaction conditions and improve its application value, *Bacillus naganoensis* pullulanase (PulA) was mutated by site-directed mutagenesis and the biochemical characteristics of the mutants were studied. The mutant PulA-N3 with mutations at asparagine 467, 492 and 709 residues was obtained. It displayed the activity maximum at 60 °C and pH 4.5 and exceeded 90% activities between 45 and 60 °C and from pH 4.0 to pH 5.5, which was improved greatly compared with wild-type PulA. Its thermostability and acidic pH stability were also remarkably improved. Its catalytic rate (*k*_cat_/*V*_max_) was 2.76 times that of PulA. In the preparation of high glucose syrup, the DX (glucose content, %) values of glucose mediated by PulA-N3 and glucoamylase reached 96.08%, which were 0.82% higher than that of PulA. In conclusion, a new pullulanase mutant PulA-N3 was successfully developed, which has high debranching activity in a wide range of temperature and pH, thereby paving the way for highly efficient starch saccharification.

## Introduction

Pullulanase is a kind of enzyme that specifically hydrolyzes α-1,6-glycosidic bonds at branch point of amylopectin. It plays an essential role in enzymatic processing of starch to either high glucose syrup or high maltose syrup^1–4^. Many different pullulanases have been biochemically identified and divided into five types (pullulanase type I, pullulanase type II, pullulan-hydrolysing enzyme type I or new pullulanase, pullulan-hydrolysing enzyme type II or isopullulanase and pullulan-hydrolysing enzyme type III) according to the differences in substrates and hydrolysis methods^5^ Currently, only type I pullulanases from *Bacillus acidopullulyticus*^6^ and *Bacillus naganoensis*^7^ are available on the market for industrial purposes.

When pullulanase is applied in the preparation of high glucose syrup, it often works with glucoamylase to promote starch saccharification efficiency. In order to meet the criteria necessary for starch saccharification, pulllulanase that has a temperature optimum of 60 °C or higher and pH optimum of 4.5 or lower is required (it is recommended by Novozymes that to operate at 61 °C and pH 4.3 for preparation of high-glucose syrup from starch). However, the wild type pullulanase from *B. naganoensis* has a temperature optimum of 55 °C and pH optimum of 5.0 with poor thermostability^8^ and its activity is limited in a narrow pH range (over 70% of its activity at pH 4.5 to 5.2)^9^, which are not well compatibility matching with that of glucoamylase. Therefore, it is valuable to obtain a pullulanase with the highest activity under more acidic pH and higher temperature with sufficient thermostability (temperature up to 60 °C) and pH stability (pH 4.0 or lower). For this purpose, several successful endeavors have been made. For example, the residue D787 lining the catalytic pocket of *B. naganoensis* pullulanase were identified and mutated, and the resulted mutant D787C maintained higher activity at temperatures over 60 °C and 90% residual activity at pH 4.0 and its enzymatic activity and specific activity were increased by 0.5-fold^10^. Using the evolutionary coupling saturation mutagenesis approach, seven residue pairs as evolutionary mutational hotspots far from the catalytic pocket of *B. naganoensis* pullulanase were predicted and the subsequent saturation mutagenesis yielded variants with enhanced catalytic activity^11^.

In the present studies, *B. naganoensis* pullulanase (PulA) was artificially evolved by site-directed mutagenesis technology. The mutants with improved starch debranching activities at higher temperature and under wider acidic pH range were developed. The values applied for starch saccharification to glucose were illustrated. To the best understanding of our knowledge, this is the first report that the debranching conditions (temperature and pH) of *B. naganoensis* pullulanase have been successfully extended and optimized.

## Materials and methods

### Strains, plasmids and primers

*Escherichia coli* JM109 and *Bacillus licheniformis* CBBD302^12^ were used as the hosts for the gene cloning and expression, respectively. The strains were grown at 37 °C and 220 rpm for 12–16 h in Luria–Bertani (LB) medium (tryptone 10.0 g/L, yeast extract 5.0 g/L, and NaCl 10.0 g/L) or at 37 °C on LB plates. When necessary, 100 µg/mL ampicillin or 20 µg/mL kanamycin was supplemented. pHY-WZX^13^ is an expressed vector in host strains from *Bacillus* genus and used to mediate the secretion and expression of pullulanase and its mutant genes. pWB-PulA carrying the pullulanase-encoded gene from *B. naganoensis* ATCC 53,909 constructed in the previous work^7^ was used as template for DNA manipulation. The sequences of oligonucleotides containing the appropriate base changes for site-directed mutagenesis are listed in Table 1 and chemically synthesized.

**Table 1:** Primers used for site-directed mutagenesis of PulA.

Primer	Nucleotide sequence (5ʹ → 3ʹ)*
467-F	GGCCCTGACGGTGTAAAGAC
467-R	GTCTTTACACCGTCAGGGCC
492-F	CGCATTTGCGAGTGTCAATG
492-R	CATTGACACTCGCAAATGCG
709-F	GGATGCTATTAAACGTGGAGTTG
709-R	CAACTCCACGTTTAATAGCATCC
PULA-F	ATAGGATCCGATGGGAACACCACA
PULA-R	CTATTTACCATCAGATGGGCTTACTT

*Underlined nucleotide sequence encoded a mutated amino acid.

### Prediction and structure analysis of pullulanase mutation sites

Based on the difference in the free energy of folding of the amino acid sequence, the mutation sites in PulA were predicted according to the method introduced in the literature^14^. The basic process for prediction of the putative mutation sites in PulA was to set the pH to 4.0 and the temperature to 60 °C in I-Mutant 2.0^14^ as the selective parameters. Each of 926 amino acid residues in PulA was inputted and the outputs were selected and grouped according to the credibility index generated by the software. According to the difference in the free energy of protein unfolding (ΔΔ*G*) formed by the amino acid residue changes in the amino acid sequence of pullulanase, the possible amino acid positions for mutation and mutated residues were further analyzed by homology modeling, structure matching and molecular docking as described below.

Pullulanase 2WAN (PDB: 2WAN)^3^ was selected as the template for PulA (GenBank: AEV53626.1) homology modeling using software SWISS-MODEL Protein-modeling Server^15–17^. Structure comparison and analysis between PulA and its mutants were performed by the program of PyMOL^18^. Maltotriose, a repeated unit joined by α-1,6 linkages in pullulan, the natural substrate of pullulanase^19^, was used as a pullulanase ligand for molecular docking by Auto Dock software^20,21^. The protein structure derived from the above homology modeling was used as the receptor structure.

### Site-directed mutagenesis of PulA

The site-directed mutagenesis of pullulanase PulA encoding gene was adopted using primer-mediated overlapping PCR method^22^. In general, partial fragments of the pullulanase-encoding gene were amplified using pWB-PulA (Wang et al., 2014) as template and two pairs of primers PULA-F and 467-R, 467-F and PULA-R (Table 1). The full-length mutant pullulanase encoding gene was then overlapped and amplified under the mediation of primers PULA-F and PULA-R (Table 1) using the mixture of the above amplified fragments as template. Other site mutations were done in the similar procedure except different pairs of primers used (Table 1). The PCR amplification condition was set as follows: Initial denaturization at 95 °C for 5 min, cycling for 30 cycles at 94 °C for 10 s, 56 °C for 1 min, and 68 °C for 3 min. The mutated pullulanase-encoding gene was cloned into pHY-WZX^13^ and transformed into *E. coli* JM109 by heat shock transformation^23^, and then into *B. licheniformis* CBBD302 according to the method as described^12^ for preparation of pullulanase mutants. The mutated sites were verified by Sanger sequencing^24^.

### Expression and purification of mutants

The transformants of *B. licheniformis* CBBD302 were grown at 37 °C and 200 rpm for 12–16 h in 50 mL LB liquid medium containing 20 μg/mL kanamycin. The culture was then inoculated at a 10% dose in 50 mL LB liquid medium in a 250 mL Erlenmeyer flask and incubated for 72 h at 37 °C and 220 rpm^12^. The supernatant was harvested by centrifugation at 8,000 rpm and 4 ℃ for 10 min and was precipitated with a saturated ammonium sulfate solution of 40–70% saturation when necessary. The enzyme was then purified by an ÄKTA Pure system using a Sephadex G-100 (10 × 500 mm) (Cytiva, Sweden) by eluting with 50 mM phosphate buffer (pH 7) at a flow rate of 0.5 mL/min. The chromatographic elution peaks were detected by the following method for the determination of the enzymatic activity of pullulanase, and the pullulanase activity parts were collected. The molecular weight and purity of the enzyme was estimated based on SDS-PAGE, selecting 10% (w/v) running gel and 5% (w/v) stacking gel^25^ using the unstained protein molecular weight marker #26,610 (Thermo Fisher Scientific, China). Protein concentration was determined by Bradford method with bovine serum albumin fraction V (Roche Diagnostics GmbH, Germany) as a reference standard^26^. The activities of PulA and the mutants were determined according to the method as described previously^7^ using pullulan (Sinopharm Chemical Reagent Co. Ltd, China) as substrate. One unit of the enzyme activity is defined as the amount of enzyme that produced one μmole of glucose reducing-sugars equivalents per minute under 60 °C and pH 4.5.

### Effects of pH and temperature on enzyme activity and kinetic parameters

The temperature optimum was determined by measuring the enzyme activity across the temperature range from 40 to 70 °C. The pH optimum was determined by assaying the enzyme activity in 20 mM of acetate-sodium acetate buffer (pH 3.5–5.8) or phosphate buffer (pH 5.8–6.5) of different pH value ranging from 3.5 to 6.5^25^. The results are shown as relative activity and the activity obtained at optimal condition was set as 100%. The thermostability was examined by measuring the enzyme activity after incubation at 45, 50, 55 and 60 °C for 0.5, 1, 1.5, 2, 2.5, and 3.0 h, respectively. The pH stability was examined by measuring the enzyme activity after adding 20 mM acetate or phosphate buffer of different pH value ranging from 3.5 to 6.5^25^ and incubated at room temperature for 1 h. The activity of untreated enzyme is taken as 100% and the percentage of relative activity is calculated relative to the highest activity.

The kinetic parameters (*K*_m_, *V*_max_, and *κ*_cat_ values) of the enzymes were determined based on the method described previously^27^, where different concentrations (0.25, 0.5, 1, 2, 4 and 8 mg/mL) of pullulan (Sinopharm Chemical Reagent Co. Ltd, China) were adopted. The values of *V*_max_ and *K*_m_ were estimated by fitting the initial rate data to the Michaelis–Menten equation using nonlinear regression.

All experiments were carried out in triplicate. The results data were represented as mean ± standard deviation of triplicate experiments.

### Preparation of glucose from starch

Preparation of glucose syrup from starch was carried out in a 100-mL reaction vessel by simulating the industrialized preparation process of starch glucose syrup. The enzyme solution comprising glucoamylase (Shandong Longkete Enzyme Co. Ltd., China) and pullulanase (PulA or PulA-N3) was added in a 100-mL reaction vessel containing 30% (w/v) maltodextrin from liquefied starch (DE value: 8, gifted by Roquette Americas). The enzyme dosage was equal to 150 U of glucoamylase and one unit of pullulanase per gram of dry maltodextrin. The saccharification process was carried out at 61 °C and pH 4.3 with 120 rpm stirring by a magnetic stirrer. During the saccharification process, samples were taken at certain time intervals and then boiled for 10 min to inactivate enzymes and the ingredients were quantitatively measured by an HPLC system (Agilent 1200 Series HPLC System) with a refractive index detector (Shodex RI-201H, Shoko Science Co. Ltd., Japan) using a Alltech Prevail Carbohydrate Es 5u column (4.6 mm IDx250 mm, 5 μm) and 65% (v/v) acetonitrile as mobile phase at a flow rate of 1 mL/min at 35°C^28^. Glucose, maltooligosaccharides and isomaltose as standards were purchased from Jiangsu Ruiyang Biotech Co., Ltd., China. The glucose contents (%, g/g) were expressed as DX value, which were calculated as the percentage of glucose content to dry matter^29^. The contents of other sugars were also measured by HPLC and calculated as the percentage of glucose content to dry matter (%, g/g).

## Results and discussion

### Selection of putative amino acid residues for mutagenesis

**Figure 1:**
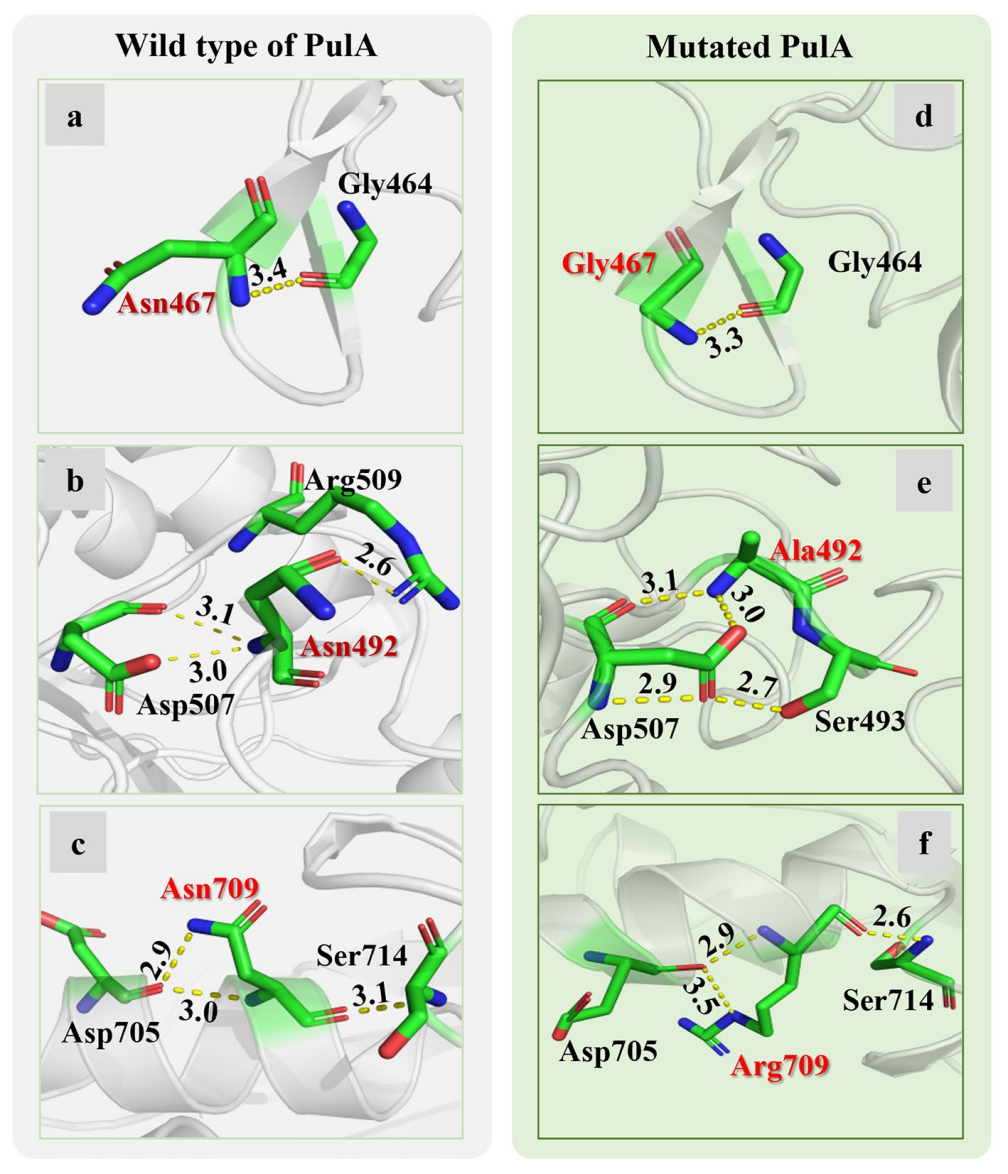
Structure diagram comparison between the wild-type and the predicted hypothetical mutation sites of PulA. The local structure of the residue N467 (**a**), N492 (**b**) and N709 (**c**) were predicted to alteration as N467G (**d**), N492A (**e**), and N709R (**f**).

The possible mutation of amino acid residues that may affect stabilities or activity in lower pH and higher temperature of PulA were first predicted using I-Mutant 2.0^14^ at pH 4.0 and 60 °C based on the difference in the free energy of protein unfolding (ΔΔ*G*) formed by the change of amino acid residues in the amino acid sequence. Total 28 amino acid residues in the PulA molecular, including S99, E100, Q108, S112, N317, N322, K327, N342, S357, N450, K453, K460, K463, N467, K469, K476, N492, N521, N523, N587, K626, S630, K665, K669, N709, N734, E758 and G776, were finally selected and might play the roles in maintaining the stability against the pH and temperature environment. It was found that asparagine residues (39.3%, 11/28) in PulA molecule were the major predicted residues. It is found that the amide group of asparagine is important in forming the side chain-backbone hydrogen bonds and fulfills an important role in stabilizing local folded structures in proteins^30^. Its deamidation is dominant pathway for protein denaturation and degradation (enzyme inactivation) and is a pH dependent process^31^. Therefore, in the present report the predicted 11 asparagine residues in PulA molecule were focused on for further investigation. By mimic 3D analysis, N467, N492 or N709 mutation might cause a significant change in the structure (Fig. 1). Alterations of N467, N492 and N709 could raise effects on the spatial structure of PulA and the putative changes produced by the mutations of N467G, N492A or N709R are shown (Fig. 1). If N467 is assumed to be changed to glycine, it extends in the peptide chain as neutral amino acid for the side chain of glycine is hydrogen bonded^32^. While N492 is assumed to be mutated to alanine, it maintains the stability of tertiary structure through hydrophobic interaction for the side chain consists of hydrophobic methyl group^33^. The mutation of N709 to arginine results in a guanidine side chain, which could facilitate the formation of more hydrogen bonds. Besides, the electric charge of arginine is more exposed to the surface of protein, which could maintain the stability of the structure by forming electrostatic interaction, and further improve the heat resistance^34^.

**Figure 2:**
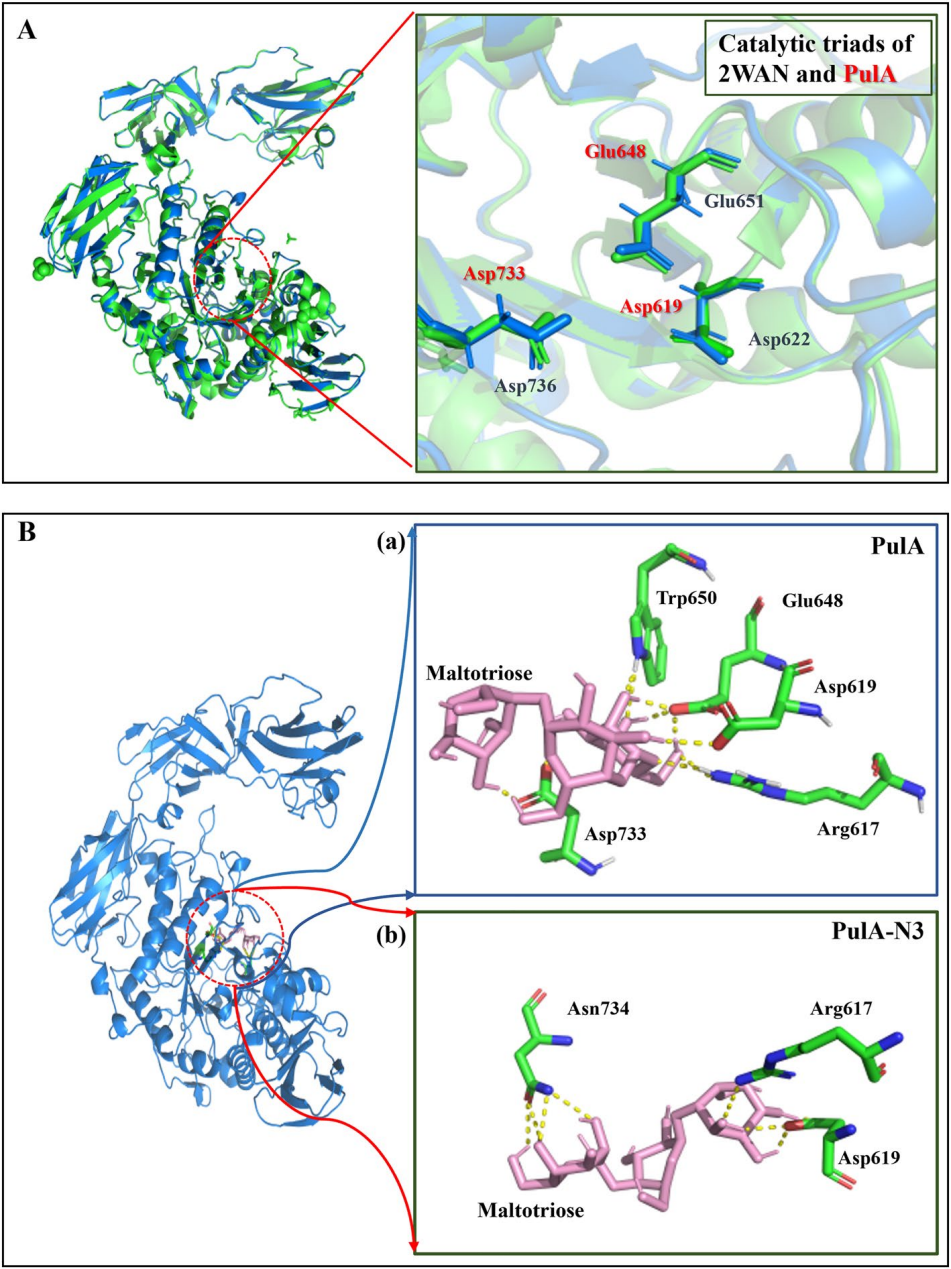
Structural schematic diagram of pullulanase. (**A**) Structural superposition of the predicted catalytic triads of PulA; B: the schematic diagram of PulA (**a**) and PulA-N3 (**b**) binding maltotriose. Pullulanase 2WAN (PDB ID: 2WAN) was used as template for homology modeling using software SWISS-MODEL Protein-modeling Server. Structure comparison and analysis of the wild-type enzyme and the mutants were performed by the program of PyMOL. Maltotriose was used as ligand for molecular docking by using Auto Dock software. The protein structure derived from the above homology modeling was used as the receptor structure. The number presented in the diagram represented the binding free energy.

To further illustrate the rational sites selected for mutation, the total binding energy of maltotriose-enzyme complex was calculated using molecular docking^20,21^. A pullulanase (PDB ID: 2WAN) with the catalytic triad composed of Asp622, Glu651 and Asp736^3^ (Turkenburg et al., 2009) was used as template for homologous modeling of PulA to predict its catalytic triad. As shown in Fig. 2A, their overall structures did not overlap completely (65.93% homology identity generated by alignment of the amino acid sequences), but their substrate binding pockets with highly conserved amino acid residues were very similar. Therefore, the catalytic triad of PulA was predicted and composed of Asp619, Glu648 and Asp733 (Fig. 2A). The schematic diagram of pullulanase binding maltotriose was then predicted by Auto Dock and the results are shown in Fig. 2B. In the substrate binding pocket for wild-type PulA, the amino acid residues directly formed hydrogen bonds with maltotriose were Arg617, Asp619, Glu648, Trp650 and Asp733. In the substrate binding pocket for the candidate mutant mutated at N467, N492 and N709 residues, the amino acid residues were changed to Arg617, Asp619 and Asn734 (Fig. 2B). They both formed eight hydrogen bonds with the substrate, however, their average binding free energy was − 3.56 and − 1.91 kcal/mol, respectively. Mutations at N467, N492 and N709 residues of PulA might considerably reduce the binding free energy.

Above analysis results strongly indicated that N467, N492 and N709 residues could play important roles in maintaining catalytic activity of PulA in lower pH and higher temperature. Therefore, they were selected for site-directed mutagenesis.

### Mutation combination of N467, N492 and N709 resulted in improved activity of *B. naganoensis* pullulanase in higher temperature and more acidic pH ranges

**Figure 3:**
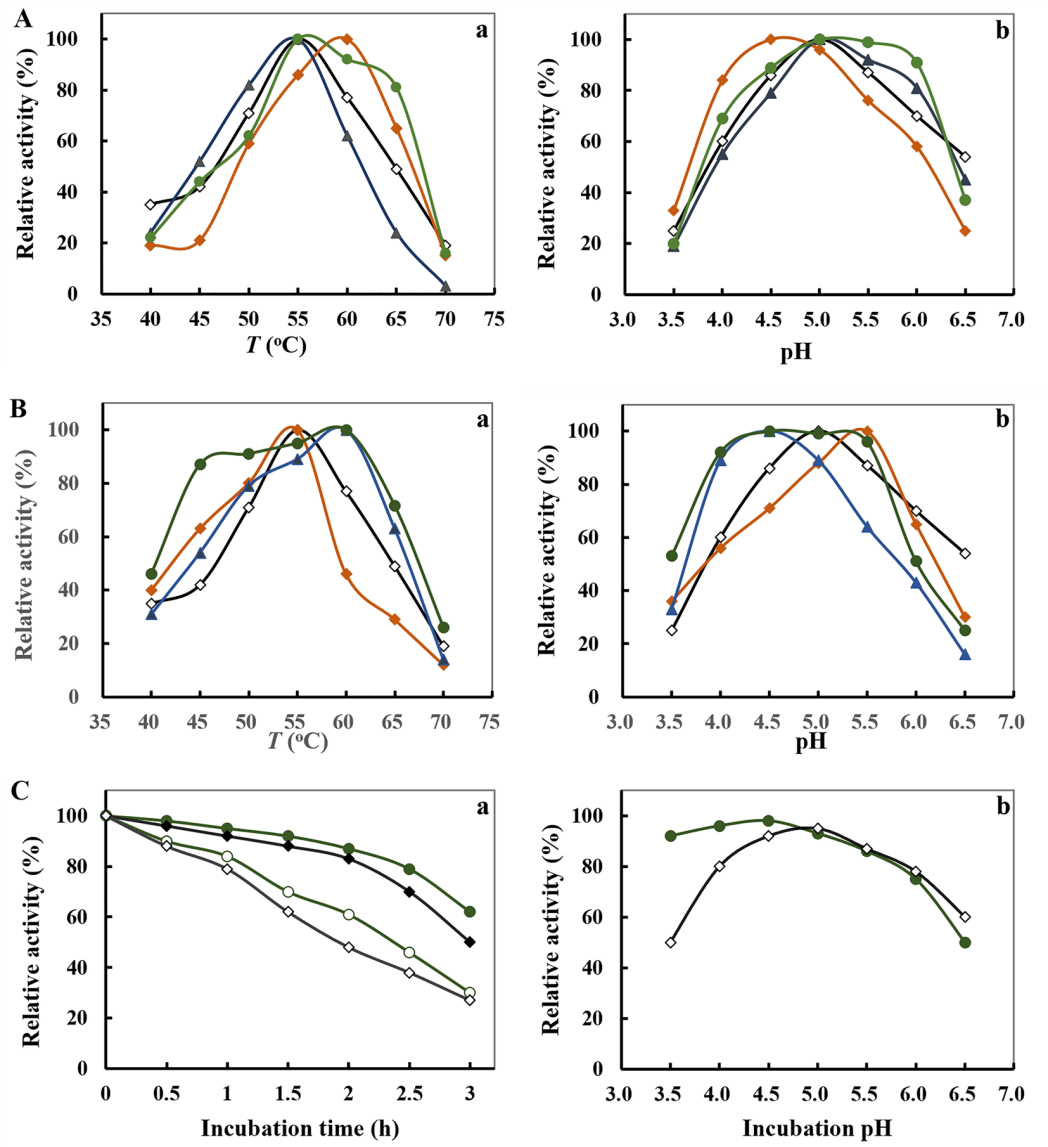
Effects of temperature (**a**) and pH (**b**) on the enzymatic activity of PulA mutants. (**A**) The temperature and pH optima of PulA (open diamond) and its single-site mutants N467G (solid diamond), N492A (solid triangle) and N709R (solid circular). (**B**) The temperature and pH optima of the multiple-site mutants PulA2-1 (N467G-N492A) (solid rectangle), PulA2-2 (N467G-709R) (solid triangle), PulA-N3 (N467G-N492A-N709R) (solid circular) and PulA (open diamond). (**C**) Thermostability and pH stability of PulA-N3 (circle) compared with PulA (diamond). The enzymes were incubated at 50 °C (solid icon) or 60 °C (open icon) for up to 3 h, or at different pH for 1 h. The residual activities were determined according to the Materials and Method section. The data were represented as average of triplicate experiments.

N467, N492 and N709 residues in PulA as predicted above were mutated using primer-mediated site-directed mutagenesis^22^. The mutants were then expressed, prepared and purified to a single band on SDS-PAGE (Supplementary Fig. S1), and their biochemical properties were analyzed. Mutation of N467G, N492A or N709R caused meaningful changes in the specific activities. Of which, the specific activity of mutant N467G was increased about 11%, that of N492A and N709R were decreased (27% and 15%, respectively) (Table 2). Meanwhile, their temperature and pH optimum were changed, however, the change tendency was of variety (Fig. 3A). Mutant N467G had the maximal activity at 60 °C and pH 4.5, which was about 5 °C higher and 0.5 pH value lower than that of wild-type PulA. The activities of mutant N709R were improved under temperature from 55 ℃ to 65 ℃ and pH from 5.0 to 6.0, although its temperature and pH optima were not much changed. No remarkable changes in the temperature and pH optima occurred in mutant N492A comparison to PulA.

**Table 2:** Specific activities (average of triplicate experiments) of PulA and mutants.

Mutants	Specific activity (U/mg pr.)	Folds of specific activity comparison to PulA
PulA	550.05	1.00
N467G	610.56	1.11
N492A	401.54	0.73
N709G	467.54	0.85
PulA-2–1	605.05	1.10
PulA-2–3	588.55	1.07
PulA-N3	660.06	1.20

According to above results, mutation of N467 noticeably changed the temperature and pH activity profile of PulA, which encouraged us to further exploit mutants with better performance through mutation combination with residues N492 and N709. Three new mutants, PulA2-1 (mutations with N467G and N492A), PulA2-2 (mutations with N467G and N709R) and PulA-N3 (mutations with N467G, N492A and N709R), were developed, and their biochemical properties were determined. Obviously and interestingly, mutant PulA-N3 performed the highest activities from 45 ℃ to 60 ℃ and from pH 4.0 to pH 5.5, which dramatically broadened the enzymatic reaction condition (Fig. 3B). Its thermostability and pH stability were also improved (Fig. 3C). Under the optimal temperature and pH, PulA-N3 performed lower *K*_m_ value and obviously increased *κ*_cat_ and *κ*_cat_/*K*_m_ values. The *κ*_cat_/*K*_m_ value of PulA-N3 was 2.76 folds of PulA (Table 3). Besides, the specific activity of PulA-N3 were increased about 20% (Table 2). The results indicated that PulA-N3 could have an enhanced catalytic rate on debranching of starch.

**Table 3:** Kinetic parameters (mean ± standard deviation of triplicate experiments) of pullulanase PulA-N3.

Enzyme	*K*_m_ (mg mL^−1^)	*k*_cat_ (s^−1^)	*k*_cat_/*K*_m_ (mL mg^−1^ s^−1^)
PulA	0.9 ± 0.07	73.5 ± 3.56	82.2 ± 3.1
PulA-N3	0.5 ± 0.05	114.7 ± 6.67	227.2 ± 18.64

### Glucose yield was improved in starch saccharification using PulA-N3

**Figure 4:**
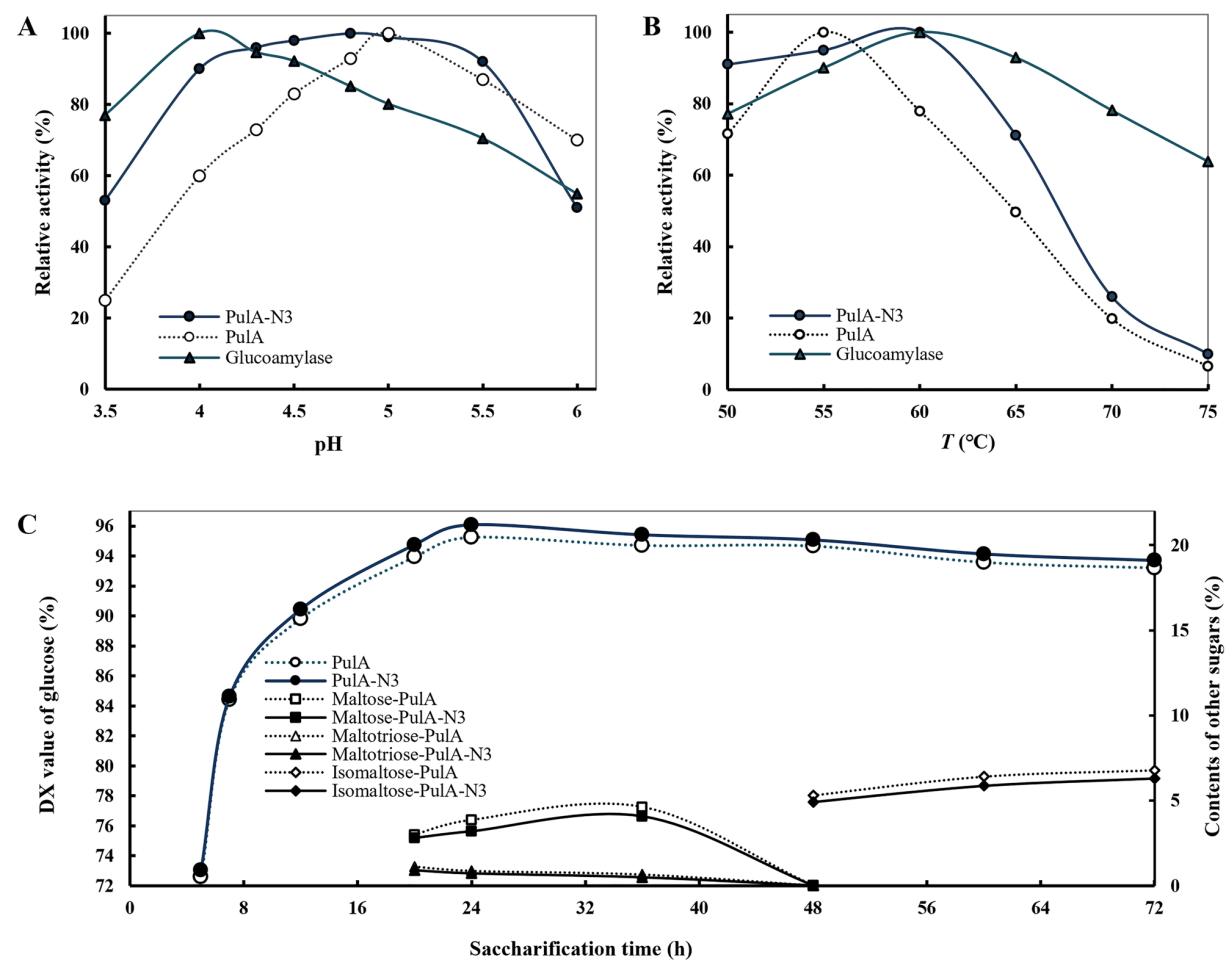
The time-course of corn starch saccharification with glucoamylase and PulA-N3. (**A**) Optimal pH for PulA-N3 and glucoamylase; (**B**) Optimal temperature for PulA-N3 and glucoamylase; (**C**) The time-course of corn starch saccharification. The saccharification experiments were carried out at 61 °C and pH 4.3 with 120 rpm stirring in 100-mL reaction vessels containing 30% (w/v) liquefied starch (DE value: 12) by adding 150 U/g (dried substrate) of glucoamylase and 1 U/g (dried substrate) of PulA-N3 or PulA. Samples were taken at certain time intervals and then boiled for 10 min to inactivate enzymes for measuring glucose content and the DX values (PulA and PulA-N3) were calculated to evaluate the degree of the saccharification by HPLC. The other sugars, including maltose (indicated as Maltose-PulA and Maltose-PulA-N3 in the figure), maltotriose (Maltotriose-PulA and Maltotriose-PulA-N3) and isomaltose (Isomaltose-PulA and Isomaltose-PulA-N3), were detected synchronously. The data were represented as average of triplicate experiments.

The pH and temperature optima of glucoamylase and pullulanase for starch saccharification were further comparatively examined and the results are summarized in Fig. 4. Glucoamylase displayed the highest activity at pH 4.0 and exhibited more than 90% of its maximum activity in the range of pH 4.0–4.5. Comparatively, mutant PulA-N3 did 96% and 90% of the highest enzyme activities at pH 4.3 and 4.0, respectively. The results clearly showed that PulA-N3 and glucoamylase shared well-matched pH optima, but PulA did not (Fig. 4A). Similar results were obtained for the optimal reaction temperature (Fig. 4B). Both Glucoamylase and PulA-N3 had the optimal temperature of 60 °C but PulA not (The optimal temperature of PulA was 55 °C and only 78% of its maximum activity at 60 °C) (Fig. 4B). The optimal reaction pH and temperature range of PulA-N3 should favor glucoamylase-led starch saccharification.

To obtain higher conversion rate of glucose on preparation of glucose syrup from starch, glucoamylase blended with pullulanase is required. The main function of glucoamylase is to hydrolyze α-1,4 glycosidic bonds to generate glucose, while pullulanase hydrolyzes α-1,6 glycosidic bonds to generate branched-chain polysaccharides, and further provides easy hydrolysis substrates for glucoamylase^35^. Since glucoamylase itself has a weak but obvious transglycosyl activity while the saccharification nearly completed in practice use, saccharification in more acidic condition is considered to reduce the amount of isomaltose formed by glucoamylase. Therefore, pullulanase mutants having the similar enzymatic properties to that of glucoamylase under more acidic condition is very likely to improve the yield from starch to glucose.

By simulating the industrial-scale process, the starch saccharification was carried out at 61 °C and pH 4.3 for 72 h using glucoamylase and PulA-N3 to hydrolyze 30% (w/v) maltodextrin. The results are summarized in Fig. 4C. In the early stages, the saccharification rate was very high. The DX values reached over 70% for 5 h incubation by glucoamylase blended with either PulA-N3 or PulA. The DX values reached 91.37% (with PulA-N3) and 89.82% (with PulA) for 12 h. After incubation for 24 h, the maximum glucose yields reached. A maximal DX value of 96.08% was achieved while PulA-N3 was used, which was increased by 0.82% comparison to that of PulA (95.26%). The content of glucose in glucose syrup is a key or even sole index for evaluating starch saccharification, which not only determines the quality and yield of high glucose syrup, but also contributes the main cost of down-stream purification. Therefore, in the starch sugar industry, whether the DX value of glucose syrup is higher than 96% or not is conventionally used as the main index for defining the qualities of both high glucose syrup and glucoamylase blends. Obviously, the use of more acidic pullulanase mutant will be more beneficial to the starch saccharification and glucose release by glucoamylase and of application potentials for formulation of high-efficiency glucoamylase blends.

Notably, the DX value of the glucose syrup showed a slow decline as the saccharification nears completion (Fig. 4C). Thus, the composition of the sugar spectrum formed during the preparation of glucose from starch was further analyzed (Fig. 4C). When the maximum DX value reached (for 24 h under this experiment conditions), there were still trace maltose and very trace maltotriose in the saccharification solution. The maltose and maltotriose were further hydrolyzed completely with the extension of saccharification time, meanwhile isomaltose was formed slowly and gradually increased over time (Fig. 4C). Since the commercially available pullulanase has no activity on isomalto-oligosaccharides^36^, optimization of saccharification endpoint and using optimized formula of glucoamylase blend may further improve the maximal DX value. Besides, other enzymes that can hydrolyze isomalto-oligosaccharides^37^ may involve in glucoamylase blend preparation.

In this report, we developed a new pullulanase mutant PulA-N3 from the wild pullulanase of *B. naganoensis* by rational site-directed mutagenesis of the predicted asparagine residues in PulA molecule. It displayed the improved activity and stability at higher temperature and under wider acidic pH ranges and the improved operating performance that well-matched with that of glucoamylase for starch saccharification (Tables 2, 3 and Figs. 3, 4). It is emphasized that the amide group of asparagine is important in forming the side chain-backbone hydrogen bonds and fulfills an important role in stabilizing local folded structures in proteins^30^. It is also well-illustrated that the deamidation of asparagine residues in proteins is dominant pathway for protein denaturation and degradation (enzyme inactivation) and is a pH dependent process^31^. Therefore, replacement of asparagine residues at specific sites of PulA with another specific amino acid may alter the side chain-backbone hydrogen forming and attenuate the deamidation. A combined mutation of N467G, N492A and N709R in PulA molecule contributed the improved activity and stability at higher temperature and low pH (Fig. 3), which indicated that asparagine residues at specific sites in pullulanases (and may also in other enzymes) were the suitable targets for artificially improving the enzyme’s activity, thermostability and acidic stability. Notably, with assistance of in silico experiments, more concise site-directed mutagenesis and less wet experiments for a desired mutant development can be obtained as the results presented in this report and many others^10,11^.

Interestingly, PulA-N3 displayed a broadened operating pH and temperature ranges, which is benefit for the process improvement of not only high glucose syrup preparation described in this report but also preparation of high maltose syrup, (iso-)malto-oligosaccharides and amylopectin as well as fruits and vegetables processing. As we know, most of natural enzymes, including pululanase, perform their maximal activity in a very narrow ranges of pH and temperature. However, the potential mechanism for these structure–function relationship should be more complex than we concerned above, which will be revealed in further studies.

Despite asparagine residues, other five amino acid residues in PulA molecule were also predicted, including the second dominant lysine residues (9/28) (K327, K453, K460, K463, K469, K476, K626, K665 and K669). It is known that lysine is the residue with the largest hydrophobic accessible surface in folded structures of proteins^38^. Site-directed mutagenesis of specific lysine residues on the enzyme surface improved the rigid β-sheet structure and thermostability of a *Bacillus* 1,3–1,4-β-glucanase^39^. Therefore, the predicted lysine residues in PulA molecule may also be the important mutation candidates to further improve its operating properties and catalytic activity under broadened pH and temperature ranges, which will be investigated in further research.

## Conclusions

The mutant PulA-N3 of *B. naganoensis* pullulanase was successfully obtained through mutation site prediction, homologous modeling and structure matching, molecular docking and site-directed mutagenesis. It displayed the improved activity and stability at higher temperature and under wider acidic pH ranges. Notably, it was more suitable for starch saccharification to produce high glucose syrup from starch in combination of glucoamylase.

## Supplementary Information

Supplementary Information.

## Data Availability

All data generated or analyzed during this study are included in this article and its supplementary information files.

## References

[1] Norman BE (1982). A novel debranching enzyme for application in the glucose syrup industry. Starch/Stärke.

[2] Bertoldo C, Antranikian G (2002). Starch-hydrolyzing enzymes from thermophilic archaea and bacteria. Curr. Opin. Chem. Biol..

[3] Turkenburg JP (2009). Structure of a pullulanase from *Bacillus acidopullulyticus*. Proteins.

[4] Wang X, Nie Y, Xu Y (2019). Industrially produced pullulanases with thermostability: Discovery, engineering, and heterologous expression. Bioresour. Technol..

[5] Nisha M, Satyanarayana T (2016). Characteristics, protein engineering and applications of microbial thermostable pullulanases and pullulan hydrolases. Appl. Microbiol. Biotechnol..

[6] Akassou M, Groleau D (2019). Advances and challenges in the production of extracellular thermoduric pullulanases by wild-type and recombinant microorganisms: A review. Crit. Rev. Biotechnol..

[7] Wang Y, Liu Y, Wang Z-X, Lu F (2014). Influence of promoter and signal peptide on the expression of pullulanase in *Bacillus subtilis*. Biotechnol. Lett..

[8] Nie Y, Yan W, Xu Y, Chen WB, Mu XQ, Wang X (2013). High-level expression of *Bacillus naganoensis* pullulanase from recombinant *Escherichia coli* with auto-induction: effect of *lac* operator. PLoS ONE.

[9] Chang M, Chu X, Lv J, Li Q, Tian J, Wu N (2016). Improving the thermostability of acidic pullulanase from *Bacillus naganoensis* by rational design. PLoS ONE.

[10] Wang X, Nie Y, Xu Y (2018). Improvement of the activity and stability of starch-debranching pullulanase from *Bacillus naganoensis* via tailoring of the active sites lining the catalytic pocket. J. Agric. Food Chem..

[11] Wang X (2020). Evolutionary coupling saturation mutagenesis: Coevolution-guided identification of distant sites influencing *Bacillus naganoensis* pullulanase activity. FEBS Lett..

[12] Niu D, Zuo Z, Shi GY, Wang Z-X (2009). High yield recombinant thermostable α-amylase production using an improved *Bacillus licheniformis* system. Microb. Cell Fact..

[13] Niu D, Wang Z-X (2007). Development of a pair of bifunctional expression vectors for *Escherichia coli* and *Bacillus licheniformis*. J. Ind. Microbiol. Biotechnol..

[14] Capriotti, E., Fariselli, P. & Casadio, R. I-Mutant2.0: predicting stability changes upon mutation from the protein sequence or structure. *Nucleic Acids Res.* **33**, W306–W310. 10.1093/nar/gki375 (2005). 10.1093/nar/gki375 PMC1160136 15980478

[15] Xiang Z (2006). Advances in homology protein structure modeling. Curr. Protein Pept. Sci..

[16] Kryshtafovych A, Fidelis K (2009). Protein structure prediction and model quality assessment. Drug Discov. Today.

[17] Zhang L (2011). Homology modeling, molecular dynamic simulation and docking studies of cyclin dependent kinase 1. J. Mol. Model..

[18] Yuan S, Chan HS, Filipek S, Vogel H (2016). PyMOL and Inkscape bridge the data and the data visualization. Structure.

[19] Leathers TD (2003). Biotechnological production and applications of pullulan. Appl. Microbiol. Biotechnol..

[20] Morris GM (2009). AutoDock4 and AutoDockTools4: Automated docking with selective receptor flexibility. J. Comput. Chem..

[21] Ferreira LG, Dos Santos RN, Oliva G, Andricopulo AD (2015). Molecular docking and structure-based drug design strategies. Molecules.

[22] Heckman KL, Pease LR (2007). Gene splicing and mutagenesis by PCR-driven overlap extension. Nat. Protoc..

[23] Maniatis, T., Sambrook, J. & Fritsch, E. F. Molecular Cloning: A Laboratory Manual. (New York, 1989).

[24] Sanger F, Nicklen S, Coulson AR (1977). DNA sequencing with chain-terminating inhibitors. Proc. Natl. Acad. Sci. USA.

[25] Zhuge, J. & Wang, Z.-X. A Lab Manual For Industrial Microbiology. (Beijing, 1994).

[26] Bradford MM (1976). A rapid and sensitive method for the quantitation of microgram quantities of protein utilizing the principle of protein-dye binding. Anal. Biochem..

[27] Malle, D. *et al*. Overexpression, purification and preliminary X-ray analysis of pullulanase from *Bacillus subtilis* strain 168. *Acta Crystallogr. Sect. F Struct. Biol. Cryst. Commun. ***62**, 381–384. 10.1107/s1744309106007901 (2006). 10.1107/S1744309106007901 PMC2222569 16582490

[28] Li S (2011). Gene cloning, heterologous expression, and characterization of a high maltose-producing α-amylase of *Rhizopus oryzae*. Appl. Biochem. Biotechnol..

[29] Linko YY, Wu XY (1993). Improvement and estimation of enzymic starch saccharification process. Biotechnol. Tech..

[30] Vasudev PG, Banerjee M, Ramakrishnan C, Balaram P (2012). Asparagine and glutamine differ in their propensities to form specific side chain-backbone hydrogen bonded motifs in proteins. Proteins.

[31] Kempkes LJ, Martens JK, Grzetic J, Berden G, Oomens J (2016). Deamidation reactions of protonated asparagine and glutamine investigated by ion spectroscopy. Rapid Commun. Mass Spectrom..

[32] Krieger F, Möglich A, Kiefhaber T (2005). Effect of proline and glycine residues on dynamics and barriers of loop formation in polypeptide chains. J. Am. Chem. Soc..

[33] Hacke M, Gruber T, Schulenburg C, Balbach J, Arnold U (2013). Consequences of proline-to-alanine substitutions for the stability and refolding of onconase. FEBS J..

[34] Sokalingam S, Raghunathan G, Soundrarajan N, Lee SG (2012). A study on the effect of surface lysine to arginine mutagenesis on protein stability and structure using green fluorescent protein. PLoS ONE.

[35] Crabb WD, Shetty JK (1999). Commodity scale production of sugars from starches. Curr. Opin. Microbiol..

[36] Niu D (2017). Highly efficient enzymatic preparation of isomalto-oligosaccharides from starch using an enzyme cocktail. Electron. J. Biotechnol..

[37] Dong Z (2018). Molecular cloning and biochemical characterization of two novel oligo-1,6-glucosidases from *Bacillus mycoides* and *Thermomyces lanuginosus*. Starch/Stärke.

[38] Lins L, Thomas A, Brasseur R (2003). Analysis of accessible surface of residues in proteins. Protein Sci..

[39] Niu C, Zhu L, Zhu P, Li Q (2015). Lysine-based site-directed mutagenesis increased rigid β-sheet structure and thermostability of mesophilic 1,3–1,4-β-glucanase. J. Agric. Food Chem..

