# Supplementary material for: Pullulanase with high temperature and low pH optima improved starch saccharification efficiency
Source: Sci Rep. 2022 Dec 19;12:21942. doi: 10.1038/s41598-022-26410-9 (PMC9763405; doi:10.1038/s41598-022-26410-9)
Supplement: Supplementary file 1 — Supplementary Information. [file 41598_2022_26410_MOESM1_ESM.pdf]

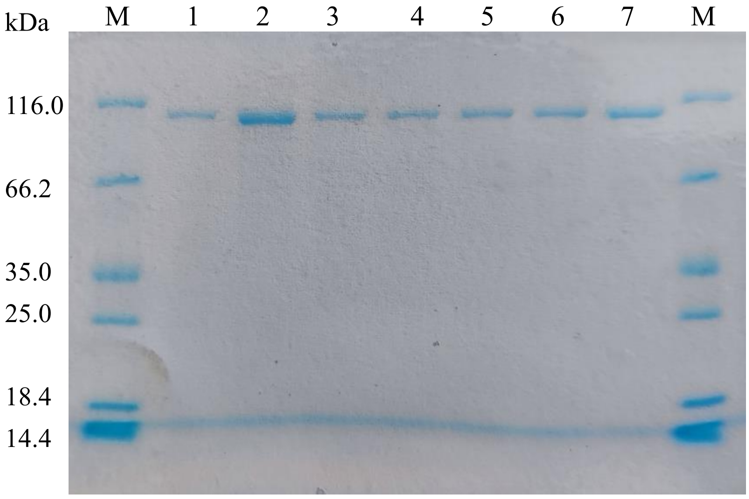

Supplementary Fig. S1. The SDS-PAGE profile of purified PulA and its mutants. From lane 1 to 7 represented PulA, mutants N467G, N492A, N709R, PulA2-1 (mutations with N467G and N492A), PulA2-2 (mutations with N467G and N709R) and PulA-N3 (mutations with N467G, N492A and N709R), respectively. M: molecular weight standard (*E. coli* β-galactosidase 116 kDa, Bovine serum albumin 66.2 kDa, Chicken egg ovalbumin 45 kDa, Porcine lactate dehydrogenase 35 kDa, *E. coli* REase Bsp98I 25 kDa, Bovine milk β-lactoglobulin 18.4 kDa, Chicken egg lysozyme 14.4 kDa).
